# A Time-Series Method for Automated Measurement of Changes in Mitotic and Interphase Duration from Time-Lapse Movies

**DOI:** 10.1371/journal.pone.0025511

**Published:** 2011-09-26

**Authors:** Frederic D. Sigoillot, Jeremy F. Huckins, Fuhai Li, Xiaobo Zhou, Stephen T. C. Wong, Randall W. King

**Affiliations:** 1 Department of Cell Biology, Harvard Medical School, Boston, Massachusetts, United States of America; 2 Department of Systems Medicine and Bioengineering, The Methodist Hospital Research Institute, Weill Cornell Medical College, Houston, Texas, United States of America; 3 Department of Translational Imaging, The Methodist Hospital Research Institute, Weill Cornell Medical College, Houston, Texas, United States of America; The Beatson Institute for Cancer Research, United Kingdom

## Abstract

**Background:**

Automated time-lapse microscopy can visualize proliferation of large numbers of individual cells, enabling accurate measurement of the frequency of cell division and the duration of interphase and mitosis. However, extraction of quantitative information by manual inspection of time-lapse movies is too time-consuming to be useful for analysis of large experiments.

**Methodology/Principal Findings:**

Here we present an automated time-series approach that can measure changes in the duration of mitosis and interphase in individual cells expressing fluorescent histone 2B. The approach requires analysis of only 2 features, nuclear area and average intensity. Compared to supervised learning approaches, this method reduces processing time and does not require generation of training data sets. We demonstrate that this method is as sensitive as manual analysis in identifying small changes in interphase or mitotic duration induced by drug or siRNA treatment.

**Conclusions/Significance:**

This approach should facilitate automated analysis of high-throughput time-lapse data sets to identify small molecules or gene products that influence timing of cell division.

## Introduction

Cell division is one of the most complex of all cellular behaviors. As a consequence, perturbations in many different cellular pathways can affect the duration of cell division, typically by delaying a specific cell cycle transition. For example, agents that damage DNA will inhibit the transition from interphase into mitosis, thereby prolonging interphase. Agents that damage the mitotic spindle prevent the transition from mitosis to interphase, thereby prolonging mitosis. Because the checkpoint mechanisms that detect these defects are highly sensitive, changes in cell cycle duration can often be observed at concentrations of perturbing agents that are below those that cause any other observable alterations in the molecular target or cellular behavior. Therefore, changes in cell cycle timing can be used as a highly sensitive detector of molecular perturbations that would be difficult to observe by other methods.

Live cell time-lapse imaging enables direct measurement of the duration of interphase and mitosis in individual cells. By using various fluorescent reporter proteins, such as H2B-GFP [1] or an engineered mitotic biosensor [2], these experiments can reveal how a perturbation affects the frequency of cell division, the fidelity of cell division [3], the duration of a specific cell cycle phase such as mitosis [4], or the timing and frequency of cell death [5]. The approach is especially powerful because each cell can be followed over time, revealing how phenotypes evolve in a time-dependent manner, and enabling different behaviors to be correlated with one another. However, a limitation of the approach is that manual analysis of time-lapse movies is tedious and time-consuming, limiting the utility of the approach for high-throughput experiments.

To address this problem, automated image analysis methods have been developed that can track cells over time and classify cells as to cell cycle stage (interphase versus mitosis or sub-phases of mitosis). However, a fully automated system for determining interphase and mitotic duration from wide-field fluorescence time-lapse movies has not been developed. Current approaches often require specialized imaging [2] or high magnification confocal images acquired in multiple planes, limiting the duration or throughput of experiments [6]. Furthermore, current analysis methods depend on supervised learning, such as support-vector machine (SVM)-based image classification [6], [7], [8], [9], [10], [11], [12], which is computationally intensive, requires extensive training, and may not be robust when applied across different cell lines or under changing experimental conditions.

Our goal in this study was to develop a fully automated method that could measure changes in interphase and mitotic duration using simple wide-field fluorescence imaging. Because most cultured cells have a cell cycle duration of more than 18 hours, we utilized a single-plane, wide-field (20×) fluorescence imaging approach that enables long-term imaging of cells. Based on these imaging parameters, we developed a time-series approach to determine cell cycle phase duration, which does not require a training data set, and is computationally rapid. The software is integrated into a complete analysis platform that is publicly available. We show that this approach can accurately determine small changes in mitosis or interphase duration induced by a variety of different perturbations.

## Results

### Identification of image features that correlate with mitotic transitions

**Figure 1:**
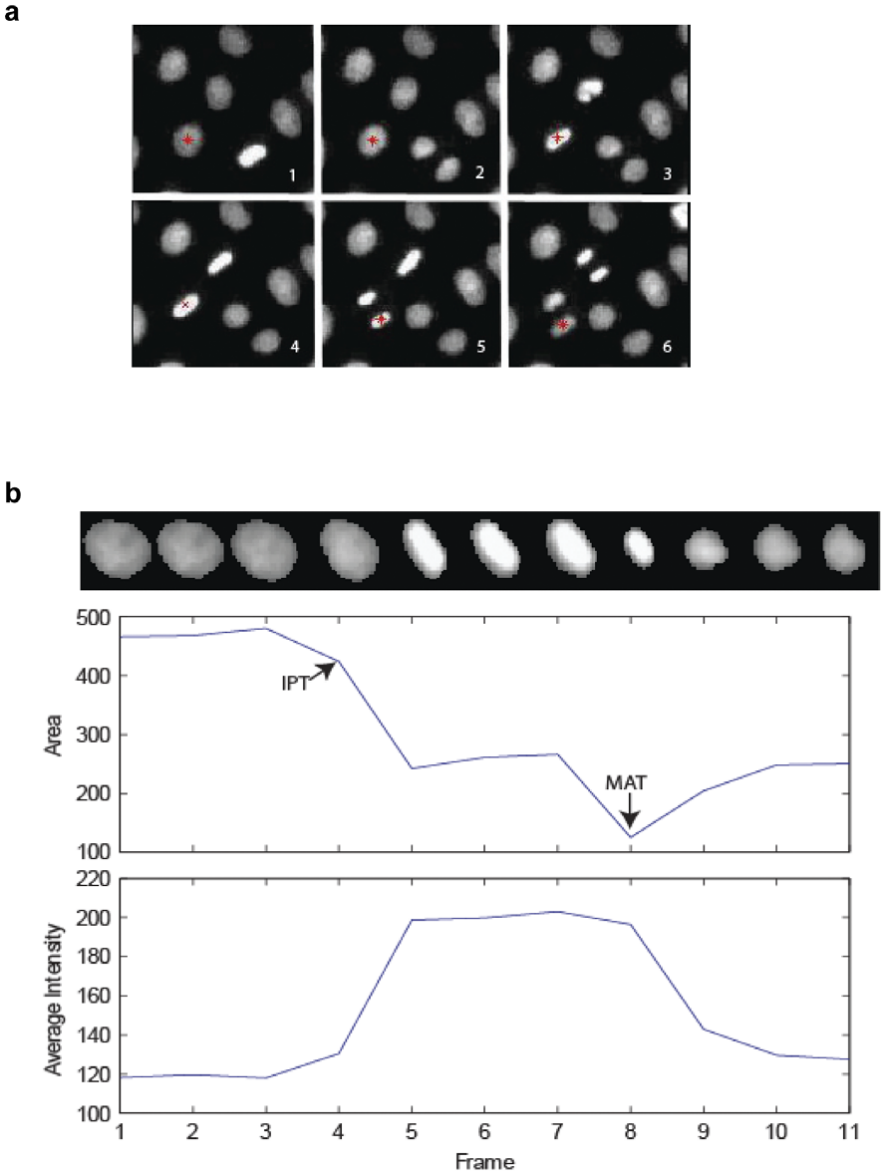
‘Area’ and ‘Average Intensity’ features are sufficient to identify the boundaries of mitosis. (a) A portion of an image of HeLa cells expressing the fluorescent marker H2B-GFP is shown. The red asterisk marks an example of a nucleus automatically tracked throughout a division. Images were acquired every 12 minutes. At the time of division (between frames 4 and 5) each daughter nucleus is tracked independently to produce a “trace” for the division. The images of the trace are then used for the time-series analysis as shown in (b). (b) An example of a trace, showing the values of average intensity (**I**) and area (**A**) graphed over time with montage of nucleus images. The interphase-prophase transition (IPT) and metaphase-anaphase transition (MAT) points are identified.

DCellIQ (Dynamic Cell Image Quantitator) is a computer package that we have developed for automated analysis of time-lapse movies of cells expressing the fluorescent nuclear marker H2B-GFP [10], [11], [13], [14]. This program automatically segments the images and identifies nuclei by local adaptive thresholding and seeded watershed segmentation with fragment merging [7], [11]. This process yields a binary image that represents the location of each nucleus, designating the region for subsequent feature extraction. Nuclei are then tracked from frame to frame by finding best matches for each nucleus based upon area, grey value histogram, XY displacement, speed, direction, shape similarity and Delaunay triangulation [7], [14], [15]. A trace is then defined as a single nucleus tracked over time, with each trace including only one daughter cell when divisions occur (Figure 1a).

In our earlier approach, features were extracted and cell cycle phase determined for each image in a trace using an SVM approach [11]. Although the SVM-based approach identified mitotic and interphase cells with high accuracy in movies on which the SVM was trained, the approach did not work as well when applied to new movies and required developing a new training dataset for each new cell line analyzed. We therefore wanted to develop an alternative approach for determination of interphase and mitotic duration that did not require retraining for each new experiment. Because nuclei undergo dramatic changes in morphology as cells enter and exit mitosis, we reasoned that a time-series based approach should allow accurately identifying key transition points. In this approach, the goal was not to individually classify each object in each image, but instead to identify key transition points based on how features of each traced object changed as a function of time.

To determine the duration of interphase and mitosis, it is necessary to identify the frame at which a cell enters mitosis (the interphase-prophase transition or IPT), and the frame at which a cell exits mitosis (defined in this case as the metaphase-anaphase transition, or MAT). These transition points were chosen because they represent the two key steps in mitotic progression that are regulated by the cell cycle machinery. We first determined which features showed reproducible and dramatic changes near these transition points. Of 211 features that can be extracted by DCellIQ [11], only changes in area (**A**) and average intensity (**I**) were reproducibly associated with the IPT and MAT (Figure 1b). **A** typically decreased at the IPT, reaching a local minimum near the MAT. In contrast, **I** typically increased rapidly near the IPT. We developed an algorithm that allowed us to first identify the IPT, and subsequently the MAT, based solely on how **A** and **I** changed over time in a given trace (see details in the Methods section and Figure S4). Mitotic duration was defined as the time between the IPT and MAT, whereas interphase duration was defined as the time between MAT of the first division in a trace and the IPT of the next division for the same trace.

### Determination of optimal imaging frequency

**Figure 2:**
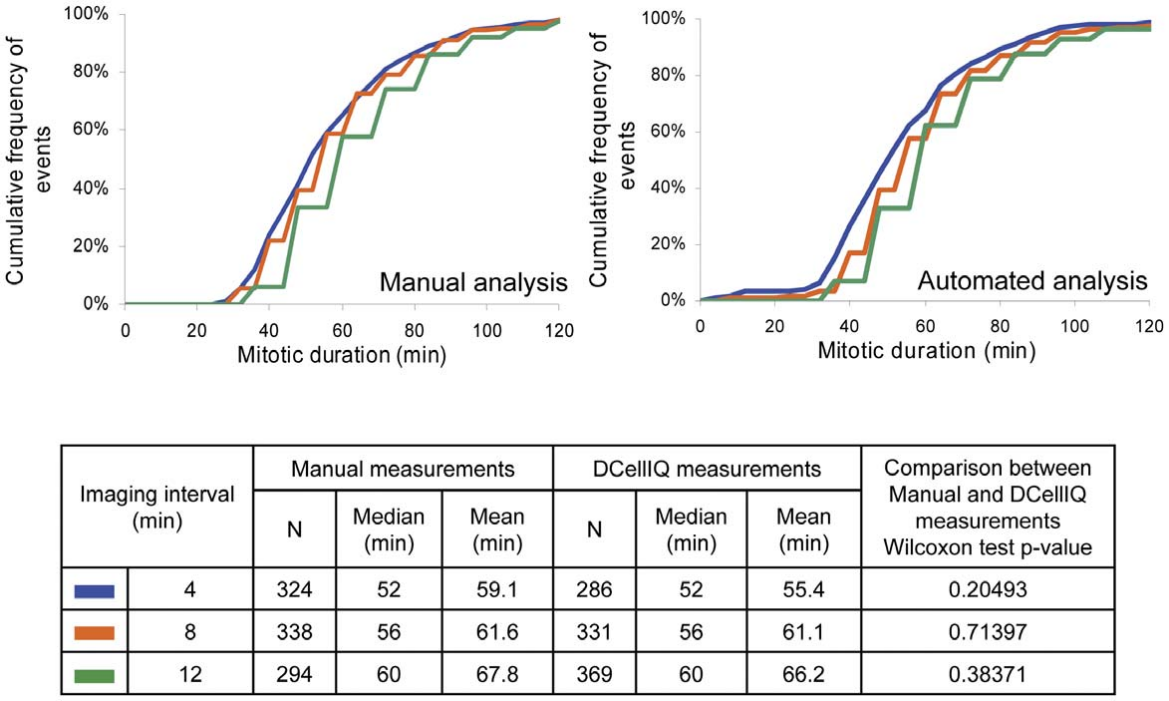
The choice of imaging frequency influences measurement of mitotic duration. HeLa H2B-GFP cells were imaged every 4 minutes for 24 hours and all images (4 min interval) or selected images (8 or 12 min intervals) were analyzed with the automated approach. For each condition 4 fields in 2 independent wells were imaged and the results for the 8 images series were combined. Half of the movies were analyzed manually for comparison. Cumulative frequency curves of events are depicted as a function of mitotic duration for the different intervals. The number of detected mitotic events (N), measured median and mean mitotic duration are provided.

A major advantage of automating the analysis of live cell imaging experiments is that one can test many more conditions in one experiment than if the data were manually analyzed. An important consideration in such experiments is the frequency of imaging. More frequent imaging provides better temporal resolution, but fewer positions can be analyzed at once, and thus fewer conditions can be tested in a given experiment. We therefore assessed how changing the frequency of image acquisition influences the ability of the time-series method to accurately measure mitotic duration as compared to manual analysis. We imaged HeLa H2B-GFP cells with an imaging interval of 4 minutes for 24 hours. This short imaging interval enables many images to be captured during mitosis (typically 12–15 during the one hour period of mitosis). Under these conditions, manual and automated analysis measured identical median mitotic durations of 52 minutes, with the automated method measuring a slightly shorter mean mitotic duration (55.4 minutes for automated analysis versus 59.1 minutes for manual analysis; Figure 2). This difference was not statistically significant (p = 0.21), but we explore the basis for this trend below. To model what would happen when the imaging frequency is decreased, we repeated manual and automated analysis on the same movies, but used every other frame or every third frame to reflect imaging intervals of 8 minutes or 12 minutes, respectively. In this case, the mean and median mitotic duration increased as imaging frequency decreased. Because manual and automated analysis provided very similar results, the change in imaging frequency, rather than the analysis method, must be responsible for the difference.

We determined that the increase in measured duration results from the fact that the interphase-prophase transition point is chosen as the last frame of interphase when early prophase is not imaged. As the imaging interval increases, the likelihood that early prophase will be missed increases, leading to a slight over-measurement of sample median and mean (See Figure S1 for example). Thus there is an inherent trade-off between imaging frequency and accuracy of measurement of mitotic duration. However, this trade-off is inherent to time-lapse imaging approaches, and is not a consequence of implementation of the automated analysis method *per se*. Based on this analysis we used frequent imaging (4 minutes) for perturbations that were expected to shorten mitotic duration, or less frequent imaging (12 minutes) for cases that were predicted to extend mitotic duration.

### TSA can identify small increases in mitotic duration

**Figure 3:**
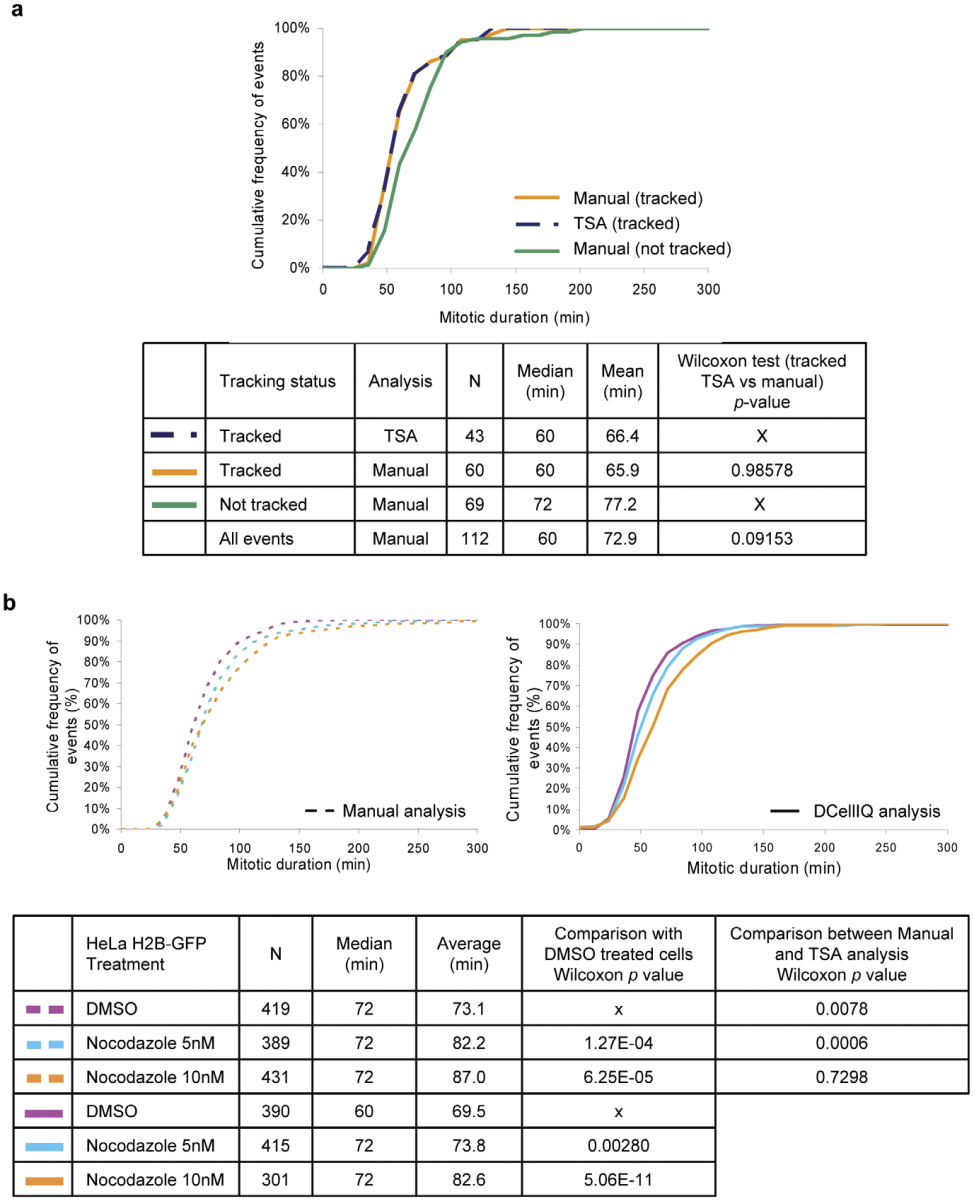
TSA detects small increases in mitotic duration when the spindle assembly checkpoint is activated by nocodazole. (a) HeLa H2B-GFP cells were imaged every 12 min for 48 hours after treatment with DMSO. Mitotic events from one movie, for nuclei that were successfully tracked, were measured by TSA (tracked successfully) and duration of these events was manually determined and both measurements were compared. Mitotic duration was also manually determined for cells that were not tracked successfully. Such cells were either not present in the imaging frame at all time throughout the movie or their trace showed potential errors of segmentation or tracking and were as a result excluded by the trace removal part of the program. Sample measurements are graphed as cumulative frequency of events as a function of mitotic duration and duration means and medians are reported. (b) HeLa H2B-GFP cells treated with indicated concentrations of nocodazole or vehicle were imaged every 12 min, for 48 hours. Half of the resulting image series were analyzed manually by identifying the duration between the first frame of mitotic entry and the first frame of anaphase for all cells in the field. All image series were analyzed by DCellIQ using TSA. For each condition, 3 fields in 3 independent wells were imaged and the results for the image series were combined. The Wilcoxon test was used to determine whether differences in mitotic duration between compared samples were statistically significant (*p*<0.05).

We first measured the accuracy of the Time Series Analysis (TSA) in measuring mitotic duration of cells that were successfully tracked by the program. We treated HeLa H2B-GFP cells with DMSO or low concentrations of the microtubule depolymerizer nocodazole and imaged the cells every 12 minutes for 48 hours (Figure 3). The TSA method identified the IPT accurately in 93% of nuclei (N = 43), compared to manual inspection. In the remaining 7% of cells, the TSA identified the IPT one frame late relative to manual analysis. The MAT was identified accurately 98% of the time. For the remaining 2% of cells, the TSA identified the transition point one frame late. This problem occurred when sister chromatids had not separated far apart enough to independently segment the two daughter nuclei. Overall, the TSA method was very accurate, as the TSA yielded a mean mitotic duration of 66.4 minutes relative to the manual measurement of 65.9 minutes, (*p* = 0.986; Figure 3a). We conclude that the time series method is very accurate in measuring mitotic duration for nuclei that are successfully tracked.

A potential limitation of the DCellIQ method is that it does not track all cells that divide during the course of the movie. Traces are removed from analysis if nuclei go out of the frame or touch the border of the frame, or if nuclei cross over one another during interphase or mitosis (see Trace Removal in Methods). During long movies necessary for determination of interphase duration, a significant proportion of cells are eliminated from analysis for these reasons. The longer the movie, or the greater the density of cells imaged, the smaller the proportion of the total number of mitotic divisions analyzed. In the current experiment lasting 48 hours, DCellIQ analyzes one-third of all mitotic events. The analysis of a subpopulation of cells by DCellIQ has the potential to introduce a selection bias into the results. In order to reveal a possible selection bias, we manually determined the mitotic duration of cells that were successfully tracked by DCellIQ and compared the measurement to that of cells that were excluded from analysis (Figure 3a). The median and mean mitotic durations were longer for cells that were not tracked successfully (72 and 77.2 minutes, respectively) compared to those that were tracked (60 and 65.9 minutes, respectively), equal to a difference of one imaging interval (12 minutes). In analyzing the underlying reason for the bias, we observed that some cells that were not tracked moved on top of adjacent interphase or mitotic cells after entering mitosis, causing the two cell nuclei to overlap or completely cross each other. This results in segmentation difficulties and makes proper tracking nearly impossible, leading to exclusion of these cells from analysis by the trace removal feature of the program. Interestingly, these cells were found to spend a longer time in mitosis, possibly because their detachment perturbs spindle formation or mitotic regulation. We compared the average duration measured by the TSA (N = 43) with the average determined from manual analysis of all cells in the movie either tracked or not tracked (N = 112), and found that the underestimation in average mitotic duration was not statistically significant for this small population (*p* = 0.0915). However, such a trend becomes statistically significant when a larger number of cells are analyzed (Figure 3b).

We next determined whether the TSA can successfully identify small increases in mitotic duration. We treated HeLa H2B-GFP cells with DMSO or low doses of nocodazole, which prolongs mitosis by activating the spindle checkpoint [16], [17]. We analyzed 9 movies per condition by TSA and manually analyzed 4 of these movies to provide sufficient data for comparison (Figure 3b). In this experiment, the manual analysis included all cells that were imaged, so that the effects of the selection bias could be evaluated. The TSA detected a statistically significant increase in mitotic duration from an average of 69.5 min for DMSO to 73.8 min for 5 nM nocodazole treatment (*p* = 0.0028). The TSA identified the increase in average mitotic duration with high statistical significance (p = 5.06×10^−11^) upon treatment with a higher dose of nocodazole (10 nM). For each condition, the TSA slightly under-measured average mitotic duration as compared to manual analysis, consistent with the bias described above. However, the selection bias did not prevent the program from detecting the increase in average mitotic duration at higher drug concentrations (15–50 nM nocodazole; Figure S2). Despite a slight under-measurement as a consequence of the selection bias, these experiments indicate that the TSA can detect subtle increases in average mitotic duration, and can accurately determine median mitotic duration.

**Figure 4:**
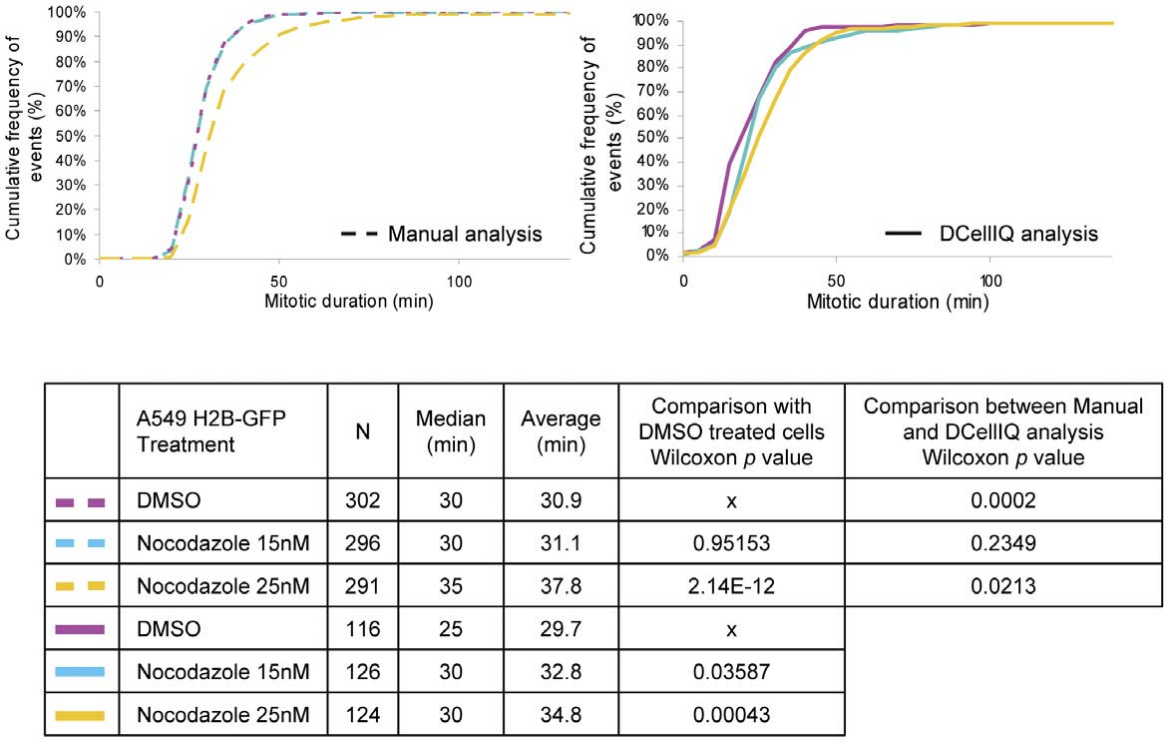
TSA detects small increases in mitotic duration when the spindle assembly checkpoint is activated by nocodazole in another cell line. A549 H2B-GFP cells treated with indicated concentrations of nocodazole or vehicle were imaged every 5 min for 48 hours. Half of the resulting image series were analyzed manually to identify the duration between the first frame of mitotic entry and the first frame of anaphase for all cells in the field. All image series were analyzed by DCellIQ using the time series approach. For each condition, 4 fields in 2 independent wells were imaged and the results for the image series were combined.

We next determined whether the same algorithm could be used to analyze changes in mitotic duration in a different cell line (A549 H2B-GFP cells, a lung cancer cell line), which has a shorter average mitotic duration (30 minutes) compared to our HeLa H2B-GFP cells. In order for DCellIQ to accurately identify mitotic transitions, we acquired images every 5 minutes, but did not otherwise alter any image analysis parameters. As for HeLa cells, the TSA detected an increase in mitotic duration upon nocodazole treatment (Figure 4). For control-treated A549 H2B-GFP cells, the time-series method accurately measured average mitotic duration as 30 minutes. Treatment with 15 nM nocodazole caused no significant change in mitotic duration but 25 nM nocodazole resulted in a significant increase in average mitotic duration to 34.8 and 37.8 min as measured by automated (*p* = 0.00043) and manual (*p* = 2.14×10^−12^) analysis, respectively. These results indicate that the time-series method can be used to detect changes in duration of mitosis in another cell line if the image sampling frequency is adjusted appropriately.

### TSA can identify decreases in mitotic duration

**Figure 5:**
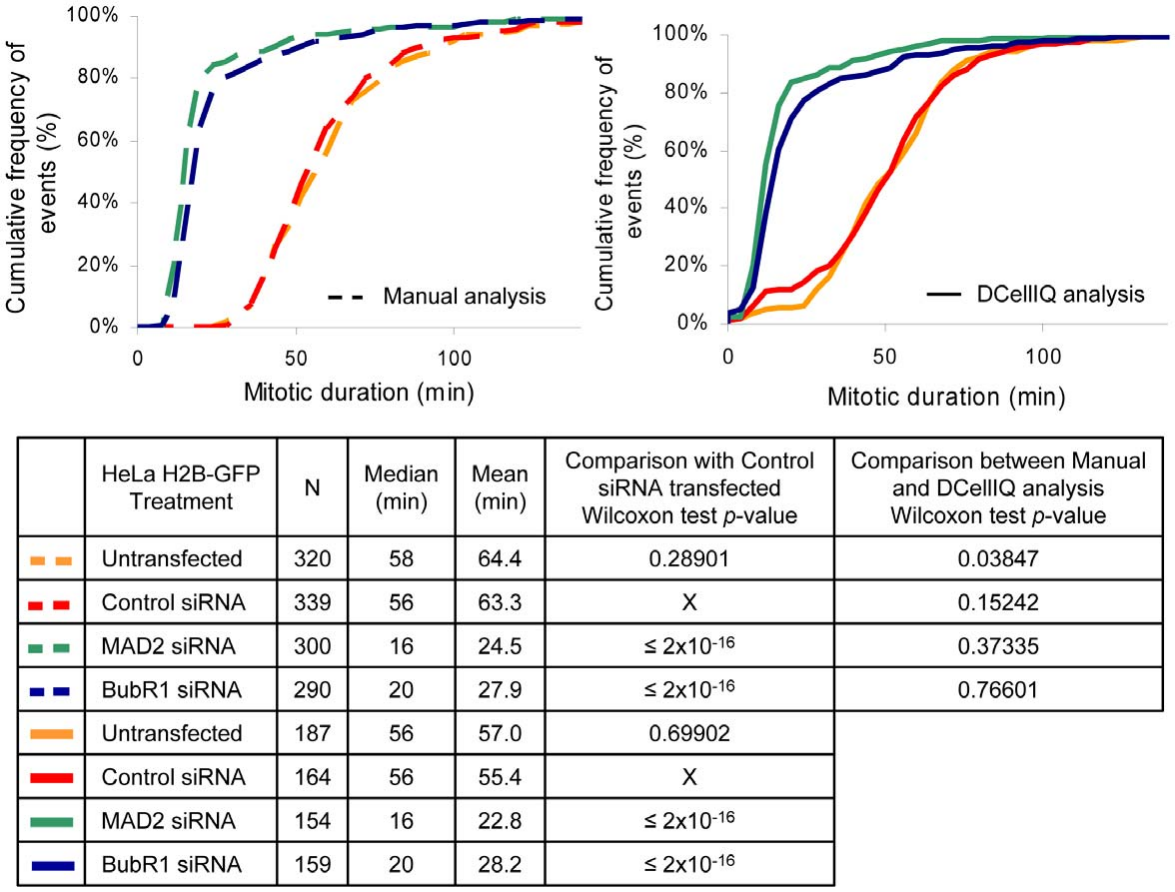
TSA detects decreases in mitotic duration when the spindle assembly checkpoint is inactivated. HeLa H2B-GFP cells were untransfected or transfected with non-targeting control, MAD2 or BubR1 siRNAs and were imaged 24 hours post-transfection, every 4 min for 48 hours. For each condition, 3 fields in 2 independent wells were imaged and the results for the 6 image series were combined. Image series were analyzed using DCellIQ and half of the movies were also analyzed manually. Cumulative frequency curves for the cell populations are provided.

To determine whether the time-series method can detect experimental perturbations that shorten mitotic duration, we analyzed mitotic timing in cells in which the spindle assembly checkpoint was inactivated by RNAi (Figure 5). HeLa H2B-GFP cells were depleted of the proteins MAD2 or BubR1 by siRNA-based transfection, which is known to shorten mitotic duration [4]. Cells were imaged for 48 hours beginning 24 hours after transfection. Images were collected every 4 minutes as cells with MAD2 or BubR1 knockdown are expected to have short mitosis in the range of 12–20 minutes [4]. For untransfected and control siRNA transfected cells, manual and TSA yielded very similar median mitotic durations of 56–58 minutes. The TSA yielded slightly shorter mean mitotic durations (57.0 and 55.4 min) compared to manual analysis (64.4 and 63.3 min) as expected based on the bias described above. In cells treated with siRNAs that target either MAD2 or BubR1, manual and time-series analysis both measured a strong reduction in median (16–20 min) and mean (23–28 min) mitotic duration (Figure 5). Interestingly, TSA did not underestimate mitotic duration in the knockdown condition, consistent with the observation that cells going through short mitosis do not round up as much and remain more attached to the culture support than cells with normal mitotic duration. Therefore, TSA can detect short mitotic events and decreases in mitotic duration, as long as cells are imaged frequently enough to acquire multiple frames during mitosis.

### TSA can measure changes in interphase duration

**Figure 6:**
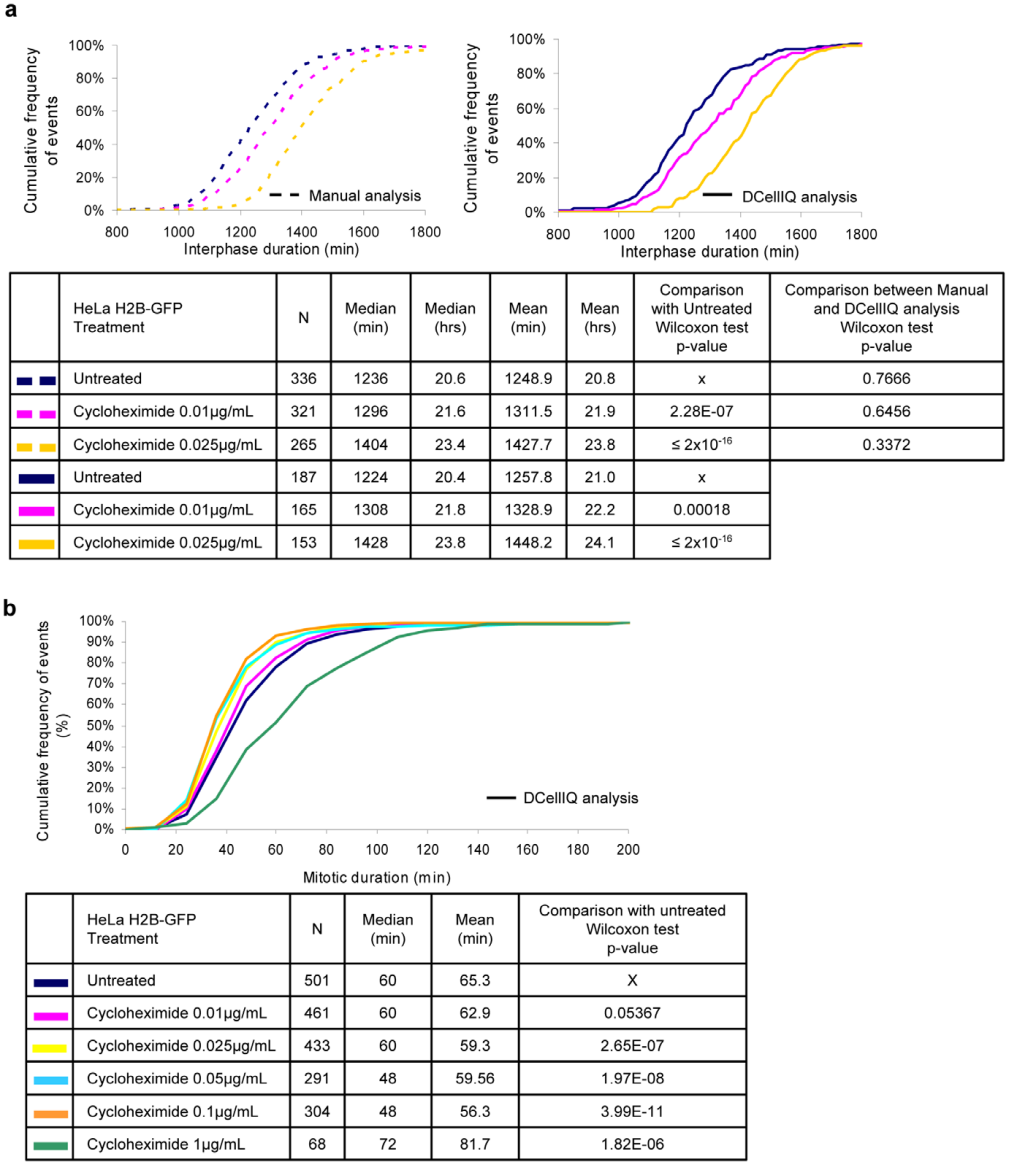
TSA detects changes in interphase and mitotic durations in response to cycloheximide treatment. HeLa H2B-GFP cells were treated with cycloheximide at indicated concentrations. Cells were imaged every 12 min for 48 hours. For each condition, 4 fields in 2 independent wells were imaged and the results for the 8 image series were combined. Image series were analyzed using DCellIQ (a and b) with the time series approach and half of them were analyzed manually (a). The origin of x-axis in graph (a) was set at 800 minutes as no interphase events had a duration shorter than 800 minutes.

Because our approach combines cell tracking over time with identification of successive mitotic divisions, it is possible to measure the duration of interphase if a cell divides at least twice during the movie. We first evaluated the effect of cycloheximide, a protein synthesis inhibitor that prevents mitotic entry at high concentrations. Low concentrations of cycloheximide (0.01 µg/mL) decrease the rate of protein synthesis in HeLa cells by 5%, whereas high concentrations (1 µg/mL) inhibit protein synthesis by 80% [18]. We treated HeLa H2B-GFP cells with cycloheximide concentrations ranging between 0.01 µg/mL and 1 µg/mL and compared the results of time-series analysis to manual analysis (Figure 6a). In untreated cells, TSA measured a median interphase duration of 20.4 h, very similar to the duration measured manually (20.6 h). At the lowest concentration of cycloheximide tested (0.01 µg/mL) TSA measured a statistically-significant increase in interphase duration (21.8 h; *p* = 0.00018), which was very similar to the value measured manually (21.6 h). At a higher dose of cycloheximide (0.025 µg/mL), TSA detected a further increase in interphase duration (23.8 h) that was again very similar to the value obtained manually (23.4 h). Higher concentrations of cycloheximide yielded a further increase in interphase duration (Figure S3). At 1 µg/mL, cycloheximide treatment induced a complete interphase arrest by the end of the movie resulting in no cell entering mitosis twice and thus interphase duration could not be determined. These findings indicate that TSA is highly accurate in determining the duration of interphase under conditions that permit cells to divide at least twice in a movie.

We also used TSA to measure how cycloheximide treatment affects mitotic duration. Interestingly, whereas low dose (0.025–0.1 µg/mL) cycloheximide increased interphase duration, it significantly shortened mitotic duration (Figure 6b; *p*≤2.65×10^−7^). At a higher concentration (1 µg/mL), cycloheximide significantly prolonged mitosis (*p* = 1.82×10^−6^). These findings highlight the utility of the TSA to identify effects that prolong one cell cycle phase while shortening another.

**Figure 7:**
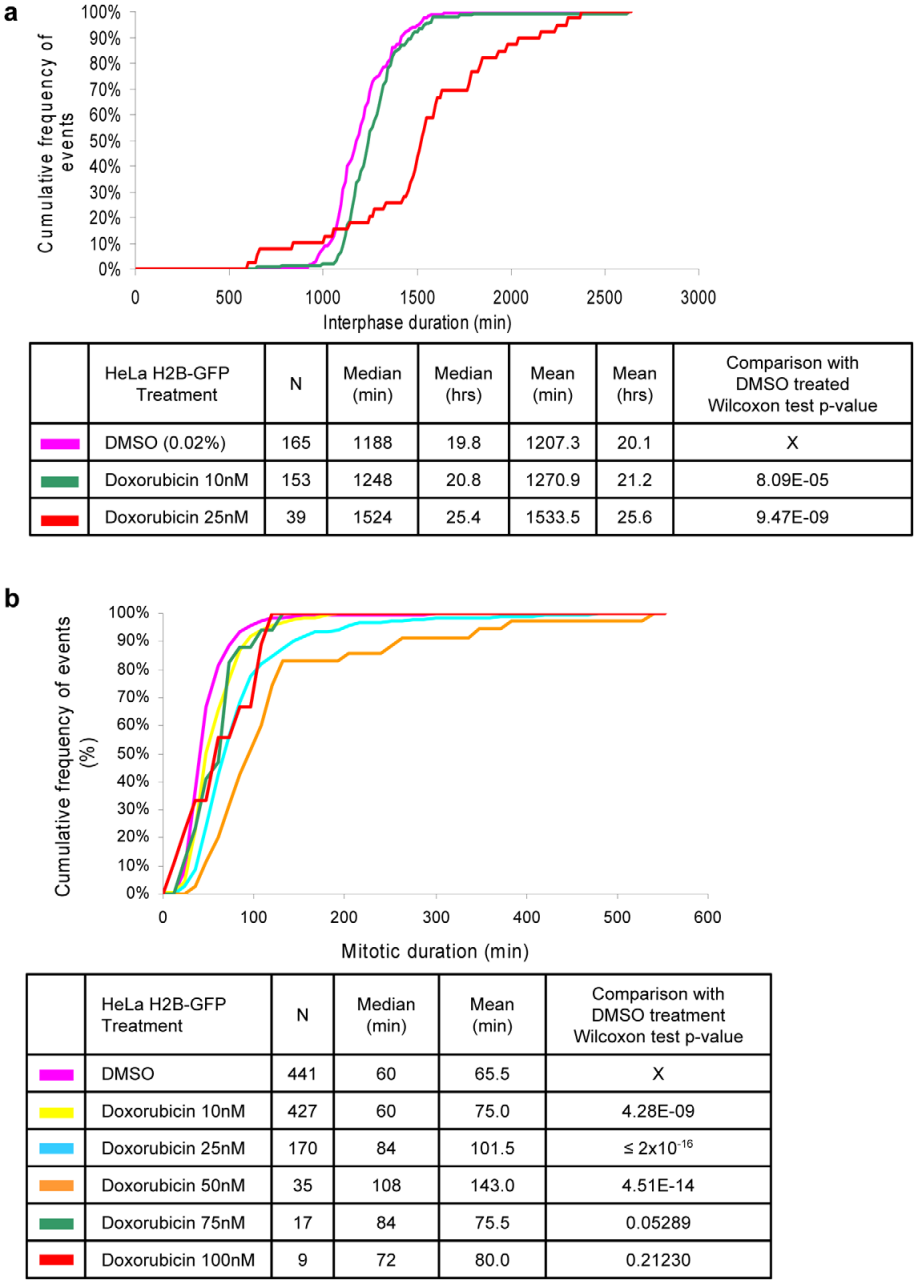
TSA detects changes in interphase duration in response to doxorubicin treatment. HeLa H2B-GFP cells were treated with doxorubicin at indicated concentrations. Cells were imaged every 12 min for 48 hours. For each condition, 4 fields in 2 independent wells were imaged and the results for the 8 image series were combined. Image series were analyzed using DCellIQ with TSA. Cumulative frequency curves of interphase (a) and mitotic (b) durations for the cell populations are provided.

As an alternative method to increase interphase duration, we treated HeLa H2B-GFP cells with doxorubicin, a topoisomerase II inhibitor which induces formation of double strand breaks and G2 phase cell cycle arrest in HeLa cells [19]. We used concentrations between 10 and 100 nM to identify drug concentrations that would delay interphase progression but still permit mitotic entry (Figure 7a). The time-series approach detected a one-hour increase in median interphase duration in cells treated with 10 nM doxorubicin, compared to DMSO treated cells (*p* = 0.00008). At higher dose (25 nM), median duration increased further to 25.4 hours. At this concentration, the number of measured interphase events decreased as fewer cells entered mitosis. At concentrations greater than 25 nM, fewer mitotic events could be observed and interphase duration could not be measured. We also measured mitotic duration in these cells. Treatment with 10–25 nM doxorubicin induced a statistically significant and dose-dependent increase in mitotic duration (Figure 7b) compared to DMSO-treated samples (*p*≤4.28×10^−9^). In conclusion, TSA enabled identification of statistically significant changes in both interphase and mitotic duration upon multiple chemical treatments.

## Discussion

Here we describe the development of a time-series based approach for automatic identification of mitotic and interphase duration from time-lapse movies of dividing cells. We show that this method can detect small changes in interphase or mitotic duration, with sensitivity comparable to changes that can be detected by manual analysis. We show that the approach can be used on a second cell line by adjusting the frequency of image acquisition to take in account differences in baseline mitotic duration.

Compared to SVM-based approaches, the time-series approach is more tolerant of changing experimental conditions, does not require extensive training, and is computationally rapid. Only two features, area and average intensity, are required to accurately identify the transition points between interphase and mitosis. These features can be easily calculated from images of relatively low magnification (20×) wide-field fluorescent imaging. The DCellIQ platform is self-sufficient, making it possible to analyze large data sets with little need for attendance by the user.

A fundamental requirement of our method is that the imaging frequency must be sufficient to capture at least three images during mitosis, or the mitotic division will not be detected. In principle, imaging at this frequency could produce phototoxicity, especially when cells are imaged for a long period of time. However, published studies that have described phototoxicity associated with long-term imaging used much longer exposures, much greater intensity, and/or more frequent illumination compared to our experiments [20], [21], [22], [23], [24]. Our imaging software and hardware settings were optimized to keep fluorescence light exposure to a minimum. These conditions produce no difference in mitotic or interphase duration compared to cells imaged by phase contrast microscopy (data not shown). The fact that our method requires only a single exposure for each time point (a single image plane) also helps limit overall exposure compared to another method that uses confocal microscopy and requires multiple planes to be acquired for each time point [6].

Most automated image analysis platforms use SVM-based approaches to classify individual images [7], [8], [9], [10], [11], [12], [13], [25]. Most SVM approaches, however, do not exploit how individual features change as a function of time in the initial step of classifying objects, potentially losing valuable information. Instead, time information is used afterwards to reconstruct how class membership of a nucleus changes as a function of time, based on input of allowed phase transitions. Furthermore, accuracy of SVM approaches may require high-resolution images that limit the number of cells that can be analyzed in a given experiment [6] or concomitant analysis of multiple markers, thereby increasing light exposure [12]. In our own experience, we have found that extensive training is required for SVM approaches to function efficiently. Training sets are very dependent on similar experimental conditions and may not be easily transferred from one cell line to another. Because TSA analyzes how extracted features change as a function of time, the approach is more tolerant of initial variation in the absolute values of these parameters between cells. For example, cells often vary in the amount of H2B-GFP protein that is expressed, leading to variation in fluorescence intensity. Because the time-series algorithm only measures how intensity changes as a function of time, the approach is less sensitive to variation in absolute levels of the reporter protein.

In our approach, the only program parameters that need to be adjusted between cell lines and conditions include the frequency of imaging (depending on the minimum mitotic length that one needs to detect), and the average nuclear size. Other parameters that can be adjusted within the program include the area threshold for initial detection of a division even, the rate of intensity change required for detecting the interphase-prophase transition, and thresholds for rates of change in area. However, we found that these parameters did not need to be altered for the time-series approach to be able to detect changes in mitotic duration in another cell line.

Analysis time becomes limiting when high-throughput imaging experiments are performed. Feature extraction is the rate-limiting step in our analysis platform. Our time-series algorithm requires only 2 features, both of which are in the geometric feature extraction category of DCellIQ, leading to a total of 11 features extracted per nucleus. In contrast, the SVM approach uses features from multiple classes, meaning that in many cases all 211 features need to be extracted. Segmentation, tracking and feature extraction of the 11 geometric features required an average of 1.2 hours per movie. Similar analysis with all 211 features for SVM processing required an average of 3.4 hours per movie. Therefore, the time-series approach is almost three times as fast as the SVM-based approach when including time needed for feature extraction.

The principal limitation of our approach is the selection bias that is imposed by the need to accurately track nuclei over long periods of time. We observed that the TSA can detect the IPT and MAT very accurately in nuclei that are successfully tracked by the program. We found that nuclei that were not successfully tracked showed a slightly longer average mitotic duration as compared to successfully tracked nuclei. However, despite this bias, DCellIQ can successfully identify small perturbations in mitotic and interphase duration, because both tracked and non-tracked cell populations respond similarly to drug treatment. Thus, although DCellIQ under-measures average mitotic duration, it accurately measures treatment effect size. Furthermore, reducing cell density, or imaging cells for a shorter period of time, will increase the fraction of cells that are accurately tracked and provide a more accurate measurement of mitotic duration in cases where such accuracy is paramount.

Other groups have developed automated or semi-automated software packages for analysis of cell division. None available for download, however, provide the functionality or ease of use that we describe here. Some packages for analysis of phase contrast movies are not fully automated, requiring partial manual analysis [20], [26]. Other software packages analyze cells expressing fluorescent markers such as H2B-GFP [9] and GFP-Histone1 [8], but no tracking function is reported by these groups. The software from Harder et al [6], [25] comes closest to our package. However, their approach requires high magnification oil-objectives (40–63×) and use of 3 to 5 confocal z-slice acquisitions, increasing light exposure and reducing the number of fields that can be imaged in a given experiment. Held et al. [12] also use an SVM approach to classify cells as interphase or sub-phases of mitosis, but the maximum duration of mitosis that is measured is 138 minutes, which may result in an underestimate of average mitotic duration under certain conditions. In contrast, our approach allows identification of mitotic events of longer duration, from 200 to 600 minutes, depending on imaging frequency. While Held et al. report high accuracy of their approach in determining mitotic duration, their manual analysis only included cells that were successfully tracked. Therefore, their method may be subject to the same type of selection bias that we report. Finally, DCellIQ is the only automated analysis platform that can automatically determine interphase duration, as other methods do not track cells for a long enough period to be able to make this measurement.

We conclude that, to our knowledge, our software package remains unique in terms of its ability to identify small changes in both mitotic and interphase duration using low fluorescence exposure imaging techniques in a platform that is convenient for the end user. We have shown that automated time-series analysis can be used to accurately measure mitotic and interphase duration with the need to extract far fewer features than needed with other methods. Our approach opens up new opportunities for time-lapse microscopy experiments that would otherwise be impossible to analyze due to the large amount of time necessary for manual analysis. Compared to fixed-cell analysis methods, automated analysis of time-lapse movies enables interphase and mitotic duration to be determined independently. Automated time-lapse analysis can therefore identify perturbations that affect both interphase and mitosis, which would be misinterpreted if a single measurement of mitotic index were performed on fixed cells.

## Materials and Methods

### Material

Cycloheximide (Calbiochem, 239764) was resuspended to 10 mg/mL in milliQ water and 0.2 µm filter sterilized. Doxorubicin (Sigma, D1515) was resuspended to 10 mM in DMSO and 0.2 µm filter sterilized. Nocodazole (Sigma, M1404) was resuspended to 10 mM in DMSO. Chemicals were aliquoted upon resuspension and stored frozen at −20°C.

### Cell Culture

Parental HeLa, A549 cell lines were purchased from ATCC. HeLa cells stably expressing H2B-GFP, previously described [3] and A549 cells were cultured in DMEM (Cell-Gro, 10-013-CV) supplemented with 10% FBS (Atlanta Biological, #S11150). To obtain stable cell lines expressing H2B-GFP, A549 cells were infected with retroviral particles containing the H2B-GFP sequence under transcriptional control of the immediate early CMV promoter. Two days after infection, the cells were selected for expression with G418 at the lowest concentration that caused death of uninfected control cells within one week. Cells were cultured at 37°C in 5% CO_2_ in a humidified environment.

### Cell Treatment

Cells were plated in sterile 24-well optical bottom plates (Greiner bio-one #662892) in 500 µL culture medium (35,000 cells per well for siRNA transfection; 45,000 cells per well for chemical treatments). After 24 hours of plating, the cells were either transfected with 200 nM of the siRNAs listed below using OligoFectamine (Invitrogen), following the manufacturer's protocol or treated with drugs at indicated concentrations. Cells were imaged 24 hours after siRNA transfection or immediately after chemical treatments at indicated time intervals. MAD2 siRNA sense strand: 5′- GGA ACA ACU GAA AGA UUG G _UU_-3′ (Dharmacon, custom sequence). BubR1 siRNA sense strand: 5′- GGA AGA AGA UCU AGA UGU A _UU_-3′ (Dharmacon, D-004101-01). Non targeting control siRNA#3 sense strand: 5′ AUG UAU UGG CCU GUA UUA G _UU_-3′ (Dharmacon, D-001210-03).

### Image Acquisition

Images were acquired with Nikon Elements (v2.3 and above) on an automated epifluorescence Ti Eclipse microscope with Perfect Focus System (Nikon Instruments Inc, USA) with a motorized Prior XYZ-plane stage, a Hamamatsu ORCA-ER digital camera and a 20× objective (PLAN APO, NA 0.75). An X-Cite light-source was used with 2 neutral density filters (ND4 and ND8) allowing 1/4^th^ and 1/8^th^ (1/32^nd^ overall) of light to go through. In addition, one field diaphragm and one aperture diaphragm present in the path of excitation light were set so as to keep the area exposed to light and the light intensity, respectively, to a minimum. The light filter set was carefully selected to strongly limit exposure to UV light (chroma filter set 49002: dichroic T495LP, excitation filter ET 470/40X, emission filter ET 525/50m). A custom designed microscope incubator set at 37°C with injection of humidified air/CO_2_ 5% gas mix was used to maintain environmental control. Cells were imaged using 200 msec exposure time and 2×2 binning resulting in an image size of 672×512 pixels. HeLa cells were imaged every 4–12 minutes, giving a total of 241–721 images per position while A549 cells were imaged every 5 minutes. Each movie was then exported in a separate folder from NIS Elements as uncompressed (12 bit) TIFF files to an 18 Terabyte network attached storage array for processing.

### Manual measurement of mitotic and interphase duration

Manual measurement of mitotic duration was performed by one experienced individual to ensure consistency. Images were analyzed in ImageJ (NIH). Mitotic duration was defined as the difference between the mitotic entry frame and the first frame showing chromatid segregation for each mitotic event visible throughout a movie. The frame of mitotic entry was defined as the first frame with nucleus showing early prophase characteristics, such as initiation of chromatin condensation. If an advanced prometaphase cell was identified as the first mitotic event, then the prior (interphase) frame was chosen as the mitotic entry to avoid underestimating mitotic duration. Each entry and exit frame as well as the mitotic duration were recorded in excel files and the data was used to calculate the median and mean event duration and to plot the cumulative frequency curves, using the Tools/Data analysis/Histogram function of Excel, for each condition tested in an experiment. Interphase duration was determined to be the time difference between the first frame of segregation of the first mitosis to the mitotic entry frame of the following mitosis. Typically half of the movies analyzed automatically were analyzed manually, to limit time of analysis to a reasonable amount while ensuring good population representation.

### Processing and Data Output

DCellIQ was run on all movies with default parameters, unless otherwise stated, with time-series or SVM phase identification as indicated, using MATLAB (The Mathworks, versions 7.5.0, 2007b to 2010b) on a Dell Precision 380 with Intel Dual P4 3.0 GHz with 3 GB of RAM. Each image series analysis data is output into files containing: duration of mitotic events, mitotic entry time in a movie for each mitotic event, cumulative mitotic duration data, duration of interphase events, interphase start time for each interphase event measured, and cumulative interphase duration data. In addition, cumulative frequency curves for mitotic duration are generated by the DCellIQ graphical user interface. DCellIQ and a user manual are freely available for download here: http://www.cbi-tmhs.org/Dcelliq/.

### Statistical analysis

For comparison of differences between independent sets of measurements, the Mann-Whitney-Wilcoxon non-parametric test was performed. Significance level was alpha = 0.05. The Mann-Whitney-Wilcoxon test relies on comparing the ranks of sample measurements of two conditions rather than comparing the sample measurement values. The measurements of both samples are ranked together. Under the null hypothesis, the distribution of the two measurement samples is the same. Under the alternative hypothesis, there would be a difference in distribution of the two measurement samples, so measurements from one sample population would precede measurements from the second sample in the distribution. The distributions in the two sample groups were considered significantly different when *p*<0.05. Statistical analysis was performed using the statistics analysis package program jmp 8.0 (SAS Institute Inc.). In some instances where the *p* value was lower than an arbitrary cutoff of 2×10^−16^, we report the values as ≤2×10^−16^.

### Segmentation, Tracking and Feature Extraction

Nuclei were segmented from the background as described previously [11]. The centroid for each nucleus was calculated in each frame. Starting from frame one, possible matches for each nucleus were identified within a 30 pixel radius. Best matches were identified based upon similarity of area, grey value histogram, XY displacement, speed, direction, shape and relationship with neighbors (Delaunay triangulation). Wavelet, geometric, moment, texture and shape descriptor features can be extracted from each nucleus identified for a total of 211 features for the SVM approach. Wang et al. fully reference the 211 features [11]. The time series involved analysis of only 11 geometry features (included in the 211 features used in the SVM approach) and Area and Intensity were the only features necessary for identifying the transitions between interphase and mitosis.

### Description of Time-Series Algorithm

The approach begins with the evaluation of the area, **A**, as a function of time (Figure S4a). Because mitotic division is associated with a decrease in **A**, the program first determines whether the trace contains any **A** values less than 230 pixels; if not, phase for the entire trace is set to interphase. In HeLa cells expressing H2B-GFP, a cutoff of 230 included 97.2% of true divisions (of 109 divisions analyzed), with a false positive rate of 4.5% (later steps in the algorithm eliminate most of these false divisions). If the trace contains any **A** value less than 230, all local minima <230 are then found using a 47 frame search window, identifying one frame containing the local minimum (Fmin) within the period window. The search window width was chosen because cells do not divide more often than once every 10 hours in 12 min interval imaging. Once Fmin is identified in each search window, the frame containing the maximum **A** value in the preceding 50 frames is identified (Fmax). Therefore, each Fmin value is associated with an Fmax, which gives an approximate indication of duration of mitosis. This approach results in a maximum potential mitotic duration of 50 frames (10 hours if imaged every 12 minutes), which may lead to underestimation of mitotic duration under conditions where cells arrest in mitosis for very long periods. In summary, the first part of the algorithm identifies a series of Fmin/Fmax pairs, with each pair representing a potential division.

The algorithm next determines the IPT using information based on either **I** or **A**, depending on the characteristics of the trace. The algorithm first calculates the 1^st^ derivative of the average intensity (***dI/dt***; abbreviated **dI**) for all frames between Fmax and Fmin. We found that there are often rapid increases (dI>30 units/frame) in **dI** at the IPT (Figure S4b). Biologically, this correlates with condensation of chromatin, producing a nucleus with greater average fluorescence intensity. If there is at least one frame between Fmax and Fmin with a **dI**>30, the algorithm chooses IPT as the frame closest to Fmin whose **dI** value exceeds 30.

However, many traces do not contain rapid increases in **I** as nuclei enter mitosis (Figure S4c), and thus an intensity-based method cannot be used to identify IPT for all traces. In these cases, the algorithm identifies the IPT based on **A**. The algorithm begins at Fmax and determines the number of frames to Fmin. If Fmax and Fmin are sequential frames, all frames are set to interphase, as this is unlikely to be a true division, because manual analysis of HeLa cell divisions indicated that no divisions were shorter than 30 minutes (2 frames) in untreated cells. The algorithm then searches forward in time from Fmax for the first frame that shows **dA**<−50, indicating a significant decrease in **A**. Biologically, this represents the decrease in nuclear area associated with chromosome condensation and the formation of the metaphase plate. Setting a threshold at this value enabled sensitive identification of mitotic divisions, but use of this criterion by itself led to a high false positive rate. We identified two reasons for identification of false positives. First, single-frame spikes in **A**, which were generally due to segmentation errors, could produce a **dA**<−50 in the subsequent frame. To address this problem, the algorithm requires that the **A** in the current frame be less than 120% of the value of the previous frame. If this criterion is not met, it suggests that an artifactual spike in **A** has occurred, and the program continues to search for another frame with a **dA**<−50. The second source of false-positive identifications is temporary decreases in **A** that are not true divisions. To eliminate these false-positives, the algorithm requires that **A** of the selected frame be substantially greater than each of the subsequent **A** values to Fmin. The algorithm uses different thresholds (either >120% or >111% of the **A** value in each subsequent frame) depending on the number of frames between Fmax and Fmin, because the rate in change of **A** is dependent on the duration of mitosis. If the current frame does not meet these criteria, the algorithm continues to search. If all of these criteria are met, however, the current frame is selected as the IPT.

The final stage of the algorithm identifies the metaphase-anaphase transition (MAT) based on analysis of **A**. In this case, MAT is defined as the first frame of anaphase. The result from the IPT algorithm is passed along to the MAT algorithm, which searches from the IPT for the **A** minimum in the following 50 frames. In most cases, this value represents the first frame of anaphase, because all subsequent frames show increases in area, reflecting decondensation of chromatin in early telophase (see Figure 1b for example). However, in some cases, we found that the local minimum corresponded to the second rather than the first frame of anaphase. These cases could be identified by comparing the value of the local minimum to the value in the previous frame.

DCellIQ detects only about half of all mitotic events before trace exclusion. Some factors that are involved in reducing the number of events obtained by automated analysis are: (1) all incomplete traces due to cells entering or exiting the frame during the movie are removed, (2) at the step of segmentation, any cell that is touching the border of the image (even by a pixel) is eliminated and is not registered as an object. For example, a cell that stays in the frame during the whole movie but touches the border just once will not result in a trace. The likelihood that a trace will be excluded from a movie for this reason increases with the length of the movie, because there is a higher likelihood that a cell will enter or exit the frame at least once if the imaging time is longer.

### Effect of Removing Incorrect Traces

Accurate measurement of interphase and mitotic duration is highly dependent on accurate tracking of a nucleus from frame to frame. The accuracy of tracking tends to decline if cell density is high, if the experiment is long, if the cells are highly motile, or if the frequency of imaging is low. One reason for this is that these conditions make it more likely that two nuclei will cross over one another during the experiment, making accurate tracking difficult. The chances of such an event occurring increase the longer the experiment is performed. Determination of interphase duration therefore places especially high demands on accurate tracking because the cells need to be followed for a long period of time. We therefore determined the accuracy of the segmentation and tracking of DCellIQ using two movies that lasted 48 hours. We found that 27% of the traces contained at least one tracking error (Figure S5a). Of these errors, 36% were a consequence of segmentation errors, 51% were due to incorrect selection of neighboring cells (due to overlapping nuclei or nuclei that approached closely to one another during the movie), and 12% were due to abnormal division of the cells (formation of more than two daughter nuclei during division). Traces containing segmentation and tracking errors show sudden increases in **A** that are not observed in correct traces. There is no biological reason for a rapid increase in **A**; however, incorrect segmentation or two nuclei crossing frequently produced a rapid increase in **A** within one or two frames. We found that incorrect traces frequently contained a large **dA** (1^st^ derivative of the **A** trace, ***dA/dt***; abbreviated **dA**) value (>130); in contrast, correct traces rarely had a **dA** value exceeding 130 (Figure S5b). At this threshold, we could remove 79% of incorrect traces while excluding only 6% of correct traces.

We analyzed the effect of implementing this approach in the analysis of an independent experiment (48 hrs movies of DMSO-treated cells analyzed in Figure 3d). Of 106 traces identified by DCellIQ, the exclusion criterion led to elimination of 66 traces. Of these 66 traces, 19 (28%) were a consequence of segmentation errors, 11 (16%) were due to abnormal cell division, and 36 (54%) were due to nuclei that overlapped during the course of the experiment. None of the remaining 40 traces contained a tracking error, indicating that this method can effectively eliminate incorrect traces. The analyses depicted in the figures in this manuscript were therefore performed using the trace removal feature of DCellIQ. A potential cost of this trace removal feature, however, is that it will also tend to exclude abnormal mitotic divisions, potentially contributing to the underestimate of mitotic duration in the population.

## Supporting Information

Figure S1 The choice of imaging frequency influences automated and manual measurement of mitotic duration equally. HeLa H2B-GFP cells were imaged every 4 minutes for 24 hours and all images (4 min interval) or selected images (8 and 12 min intervals) were analyzed manually and with the automated approach. An example of changes in Area and average intensity for a nucleus are presented for 4, 8 and 12 min analysis (a, b and c, respectively). Mitotic entry and anaphase were determined using the corresponding nucleus images (arrows in a–d).
(PDF)

Figure S2 DCellIQ detects dose-dependent increases in mitotic duration upon treatment with higher doses of nocodazole. HeLa cells expressing H2B-GFP were treated as indicated and imaged as in Figure 3. Image series were analyzed using DCellIQ with the time series approach. Cumulative frequency curves of mitotic duration for the cell populations are provided. The number of events in each sample (N), event duration median and mean as well as *p* values for the Mann Whitney Wilcoxon statistical comparisons are provided.
(PDF)

Figure S3 The TSA approach detects dose-dependent increases in interphase duration upon treatment with higher doses of cycloheximide. HeLa H2B-GFP cells were treated with indicated concentrations of cycloheximide or untreated. Cumulative frequency curves of interphase duration for the cell populations are provided. The number of events in each sample (N), event duration median and mean as well as *p*-values for the Mann Whitney Wilcoxon statistical comparisons are provided.
(PDF)

Figure S4 Time Series Analysis algorithm. (**a**) Flow chart displaying the algorithm used to identify interphase-prophase transition (IPT) and metaphase-anaphase transition (MAT) points using only area and intensity features. See Methods for detailed description. (**b**) Example of a trace in which changes in intensity are used to identify the IPT. (**c**) Example of a trace in which changes in intensity are not sufficient to identify the IPT. In this instance, changes in area are used to identify the IPT. Parameters that can be modified by the user are indicated by a red number. The parameter order corresponds to the order they appear in the program input window. The parameters are: (1) Minimum area (A) threshold for primary identification of divisions in a trace (pixels); (2) Search window duration around local minima frames (included) in which to find area minima below the Area threshold; (3) Number of preceding frames in which to find the highest A value; (4) Intensity threshold above which to use the intensity data to identify the Interphase to Prophase Transition (IPT) frame (5) Change of area threshold for primary detection of entry into prophase (pixels); (6) Change of area threshold for refined detection of entry into prophase (% of previous frame), (7)Threshold number of frames between current frame and frame with lowest A (Fmin) for subsequent Area change analysis, (8) Change of area threshold for refined detection of entry into prophase (% of area from any subsequent frame until frame Fmin); (9) Change of area threshold for refined detection of entry into prophase (% of area from any subsequent frame until frame Fmin); (10) Number of subsequent frames in which to identify the frame with lowest area; (11) Change in area threshold for MAT frame decision (% of area from previous frame).
(PDF)

Figure S5 Correct and incorrect traces can be separated by a threshold in change in Area (*dA*). (**a**) Tracking accuracy of HeLa H2B-GFP traces (n = 173). Some traces contained multiple types of errors. (**b**) Cumulative percent of maximum slope of area (*d* **A**) for each nucleus tracked over all frames, manually categorized into correct and incorrect traces. Based on this analysis, 95% of correct traces contain a *d* **A** value less than 130. In contrast, 80% of incorrect traces contain a *d* **A** value greater than 130. By using this threshold, 80% of incorrect traces can be removed while removing only 5% of correct traces.
(PDF)

## References

[1] Kanda T, Sullivan KF, Wahl GM (1998). Histone-GFP fusion protein enables sensitive analysis of chromosome dynamics in living mammalian cells.. Curr Biol.

[2] Jones JT, Myers JW, Ferrell JE, Meyer T (2004). Probing the precision of the mitotic clock with a live-cell fluorescent biosensor.. Nat Biotechnol.

[3] Shi Q, King RW (2005). Chromosome nondisjunction yields tetraploid rather than aneuploid cells in human cell lines.. Nature.

[4] Meraldi P, Draviam VM, Sorger PK (2004). Timing and checkpoints in the regulation of mitotic progression.. Dev Cell.

[5] Gascoigne KE, Taylor SS (2008). Cancer cells display profound intra- and interline variation following prolonged exposure to antimitotic drugs.. Cancer Cell.

[6] Harder N, Mora-Bermudez F, Godinez WJ, Wunsche A, Eils R (2009). Automatic analysis of dividing cells in live cell movies to detect mitotic delays and correlate phenotypes in time.. Genome Res.

[7] Chen X, Zhou X, Wong ST (2006). Automated segmentation, classification, and tracking of cancer cell nuclei in time-lapse microscopy.. IEEE Trans Biomed Eng.

[8] Gambe AE, Ono RM, Matsunaga S, Kutsuna N, Higaki T (2007). Development of a multistage classifier for a monitoring system of cell activity based on imaging of chromosomal dynamics.. Cytometry A.

[9] Neumann B, Held M, Liebel U, Erfle H, Rogers P (2006). High-throughput RNAi screening by time-lapse imaging of live human cells.. Nat Methods.

[10] Wang M, Zhou X, King RW, Wong ST (2007). Context based mixture model for cell phase identification in automated fluorescence microscopy.. BMC Bioinformatics.

[11] Wang M, Zhou X, Li F, Huckins J, King RW (2008). Novel cell segmentation and online SVM for cell cycle phase identification in automated microscopy.. Bioinformatics.

[12] Held M, Schmitz MH, Fischer B, Walter T, Neumann B (2010). CellCognition: time-resolved phenotype annotation in high-throughput live cell imaging.. Nat Methods.

[13] Yan P, Zhou X, Shah M, Wong ST (2008). Automatic segmentation of high-throughput RNAi fluorescent cellular images.. IEEE Trans Inf Technol Biomed.

[14] Li F, Zhou X, Ma J, Wong ST (2010). Multiple Nuclei Tracking Using Integer Programming for Quantitative Cancer Cell Cycle Analysis.. IEEE Trans Med Imaging.

[15] Nath SK, Palaniappan K, Bunyak F (2006). Cell segmentation using coupled level sets and graph-vertex coloring.. Med Image Comput Comput Assist Interv Int Conf Med Image Comput Comput Assist Interv.

[16] Shi J, Orth JD, Mitchison T (2008). Cell type variation in responses to antimitotic drugs that target microtubules and kinesin-5.. Cancer Res.

[17] Gupta RS (1985). Species-Specific Differences in Toxicity of Antimitotic Agents Toward Cultured Mammalian Cells.. J Natl Cancer Inst.

[18] Kong YW, Cannell IG, de Moor CH, Hill K, Garside PG (2008). The mechanism of micro-RNA-mediated translation repression is determined by the promoter of the target gene.. Proc Natl Acad Sci U S A.

[19] Vogel C, Hager C, Bastians H (2007). Mechanisms of mitotic cell death induced by chemotherapy-mediated G2 checkpoint abrogation.. Cancer Res.

[20] Davis PJ, Kosmacek EA, Sun Y, Ianzini F, Mackey MA (2007). The large-scale digital cell analysis system: an open system for nonperturbing live cell imaging.. J Microsc.

[21] de With A, Greulich KO (1995). Wavelength dependence of laser-induced DNA damage in lymphocytes observed by single-cell gel electrophoresis.. J Photochem Photobiol B.

[22] Dixit R, Cyr R (2003). Cell damage and reactive oxygen species production induced by fluorescence microscopy: effect on mitosis and guidelines for non-invasive fluorescence microscopy.. Plant J.

[23] Godley BF, Shamsi FA, Liang FQ, Jarrett SG, Davies S (2005). Blue light induces mitochondrial DNA damage and free radical production in epithelial cells.. J Biol Chem.

[24] Hockberger PE, Skimina TA, Centonze VE, Lavin C, Chu S (1999). Activation of flavin-containing oxidases underlies light-induced production of H2O2 in mammalian cells.. Proc Natl Acad Sci U S A.

[25] Harder N, Eils R, Rohr K (2008). Automated classification of mitotic phenotypes of human cells using fluorescent proteins.. Methods Cell Biol.

[26] Debeir O, Megalizzi V, Warzee N, Kiss R, Decaestecker C (2008). Videomicroscopic extraction of specific information on cell proliferation and migration in vitro.. Exp Cell Res.

