# Supplementary figures and images for: A Time-Series Method for Automated Measurement of Changes in Mitotic and Interphase Duration from Time-Lapse Movies
Source: PLoS One. 2011 Sep 26;6(9):e25511. doi: 10.1371/journal.pone.0025511 (PMC3180452; doi:10.1371/journal.pone.0025511)

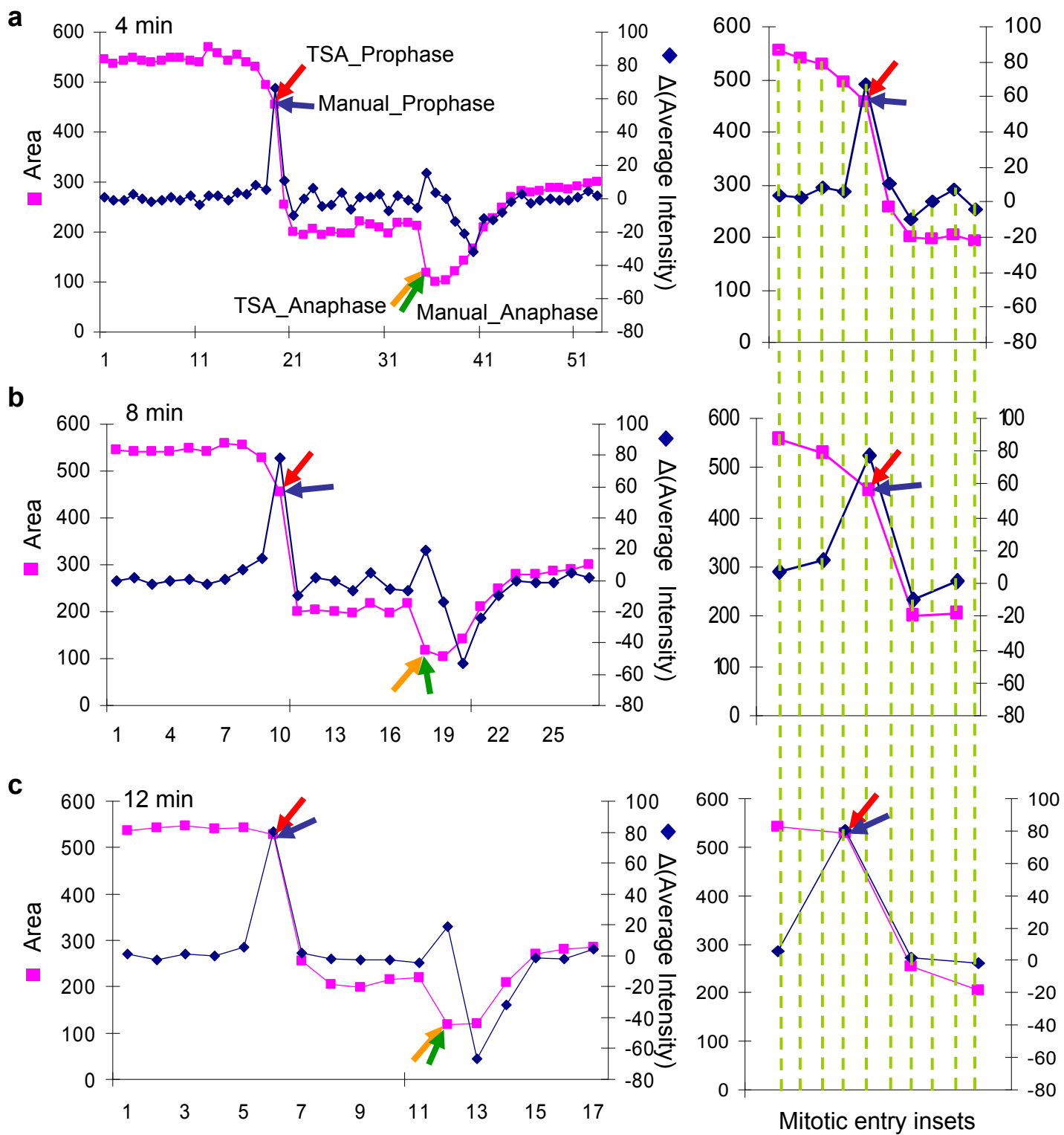

**Figure S1.**

**d**

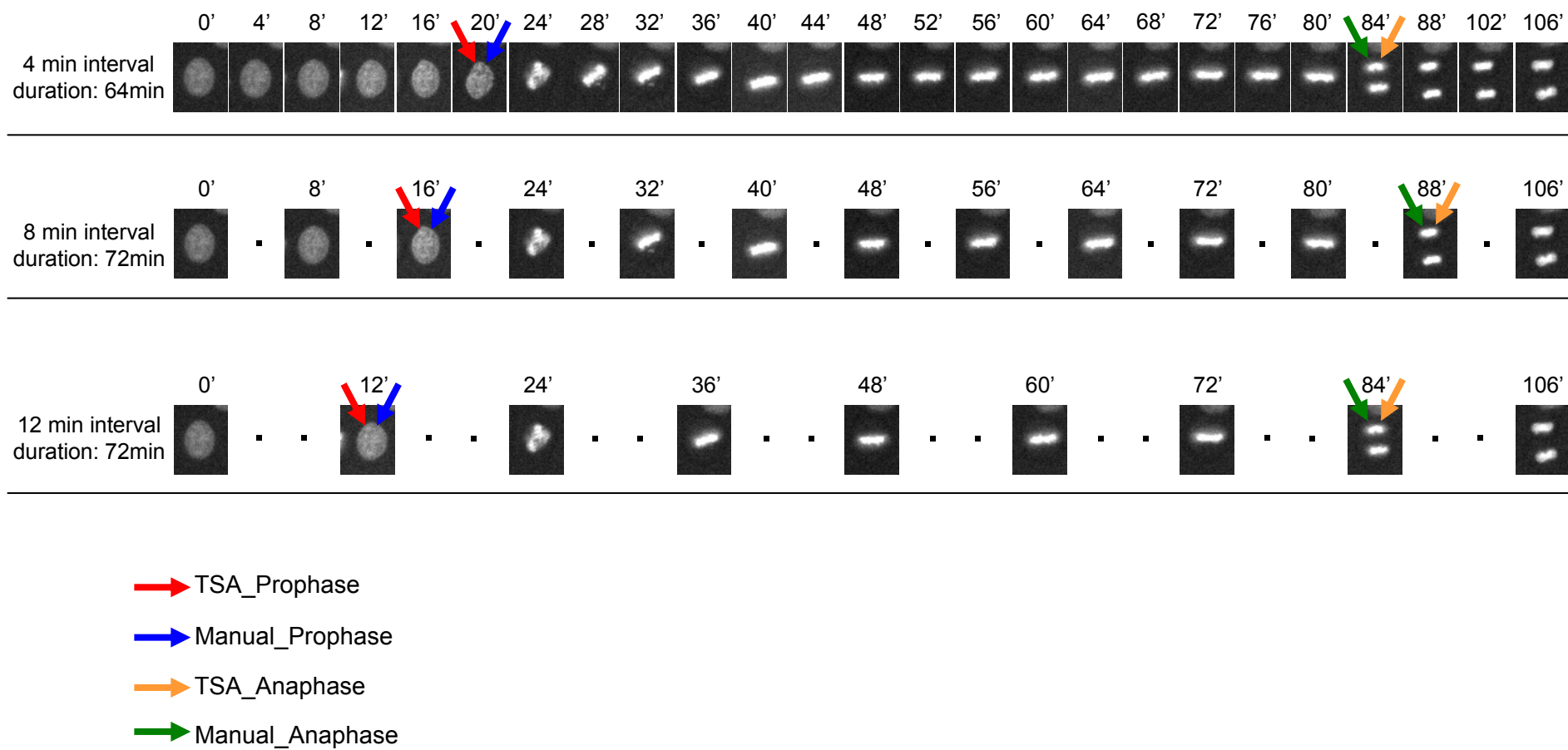

**Figure S1.**

Supplement: Figure S1 — The choice of imaging frequency influences automated and manual measurement of mitotic duration equally. HeLa H2B-GFP cells were imaged every 4 minutes for 24 hours and all images (4 min interval) or selected images (8 and 12 min intervals) were analyzed manually and with the automated approach. An example of changes in Area and average intensity for a nucleus are presented for 4, 8 and 12 min analysis (a, b and c, respectively). Mitotic entry and anaphase were determined using the corresponding nucleus images (arrows in a–d). (PDF) [file pone.0025511.s001.pdf]

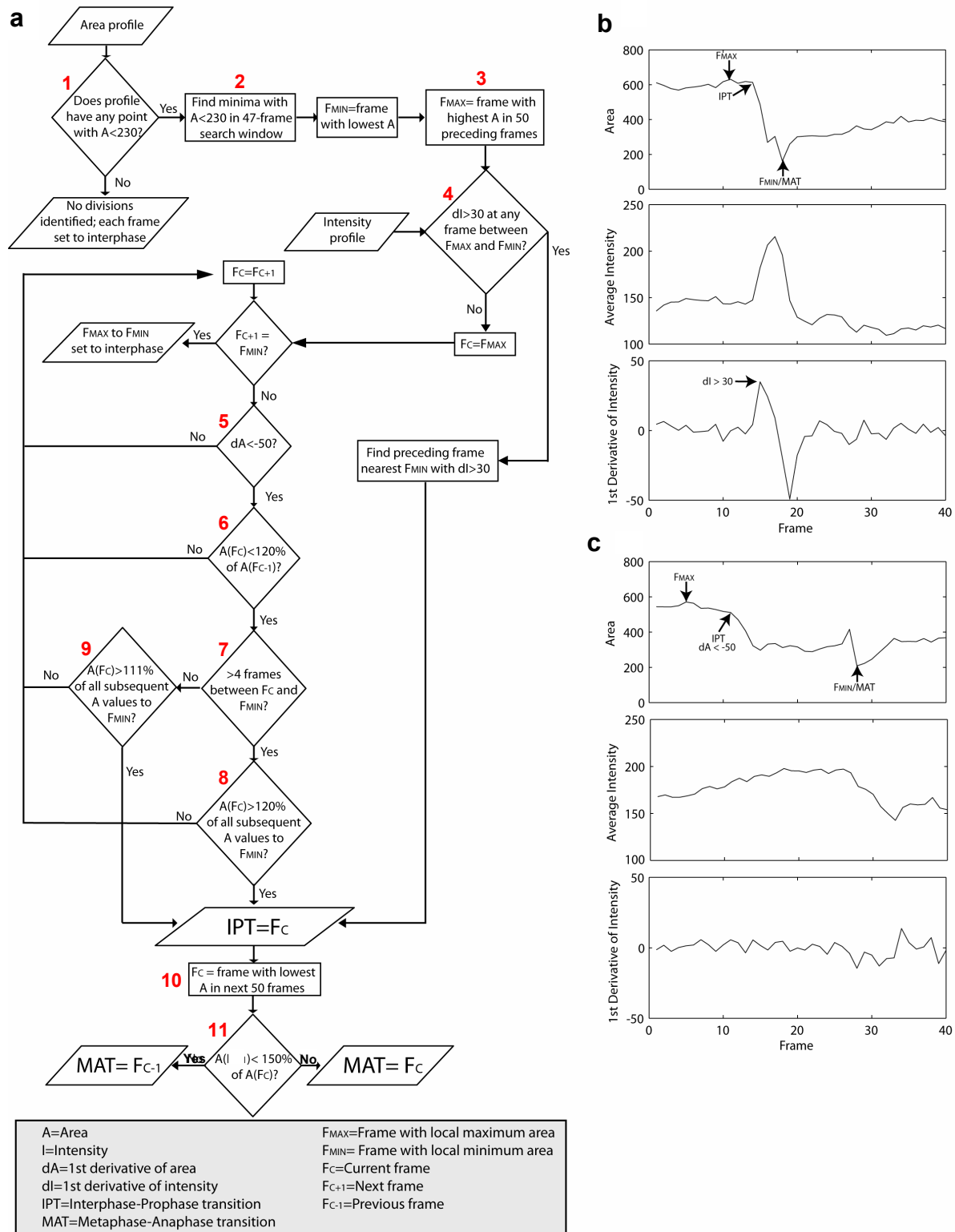

**Figure S4.**

Supplement: Figure S4 — Time Series Analysis algorithm. (a) Flow chart displaying the algorithm used to identify interphase-prophase transition (IPT) and metaphase-anaphase transition (MAT) points using only area and intensity features. See Methods for detailed description. (b) Example of a trace in which changes in intensity are used to identify the IPT. (c) Example of a trace in which changes in intensity are not sufficient to identify the IPT. In this instance, changes in area are used to identify the IPT. Parameters that can be modified by the user are indicated by a red number. The parameter order corresponds to the order they appear in the program input window. The parameters are: (1) Minimum area (A) threshold for primary identification of divisions in a trace (pixels); (2) Search window duration around local minima frames (included) in which to find area minima below the Area threshold; (3) Number of preceding frames in which to find the highest A value; (4) Intensity threshold above which to use the intensity data to identify the Interphase to Prophase Transition (IPT) frame (5) Change of area threshold for primary detection of entry into prophase (pixels); (6) Change of area threshold for refined detection of entry into prophase (% of previous frame), (7)Threshold number of frames between current frame and frame with lowest A (Fmin) for subsequent Area change analysis, (8) Change of area threshold for refined detection of entry into prophase (% of area from any subsequent frame until frame Fmin); (9) Change of area threshold for refined detection of entry into prophase (% of area from any subsequent frame until frame Fmin); (10) Number of subsequent frames in which to identify the frame with lowest area; (11) Change in area threshold for MAT frame decision (% of area from previous frame). (PDF) [file pone.0025511.s004.pdf]

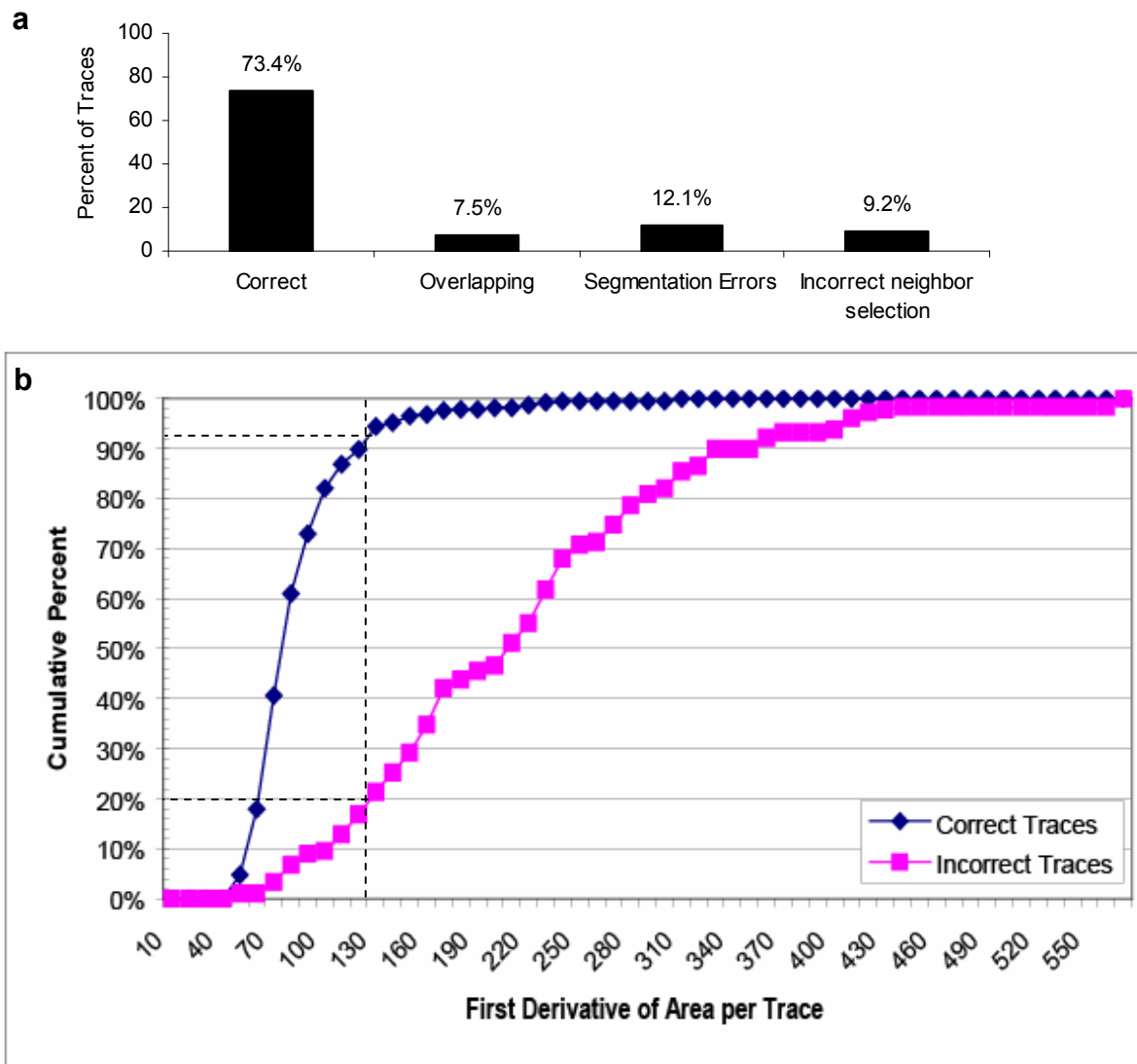

Figure S5.

Supplement: Figure S5 — Correct and incorrect traces can be separated by a threshold in change in Area (dA). (a) Tracking accuracy of HeLa H2B-GFP traces (n = 173). Some traces contained multiple types of errors. (b) Cumulative percent of maximum slope of area (d A) for each nucleus tracked over all frames, manually categorized into correct and incorrect traces. Based on this analysis, 95% of correct traces contain a d A value less than 130. In contrast, 80% of incorrect traces contain a d A value greater than 130. By using this threshold, 80% of incorrect traces can be removed while removing only 5% of correct traces. (PDF) [file pone.0025511.s005.pdf]
