# Supplementary material for: A Time-Series Method for Automated Measurement of Changes in Mitotic and Interphase Duration from Time-Lapse Movies
Source: PLoS One. 2011 Sep 26;6(9):e25511. doi: 10.1371/journal.pone.0025511 (PMC3180452; doi:10.1371/journal.pone.0025511)
Supplement: Figure S2 — DCellIQ detects dose-dependent increases in mitotic duration upon treatment with higher doses of nocodazole. HeLa cells expressing H2B-GFP were treated as indicated and imaged as in Figure 3. Image series were analyzed using DCellIQ with the time series approach. Cumulative frequency curves of mitotic duration for the cell populations are provided. The number of events in each sample (N), event duration median and mean as well as p values for the Mann Whitney Wilcoxon statistical comparisons are provided. (PDF) [file pone.0025511.s002.pdf]

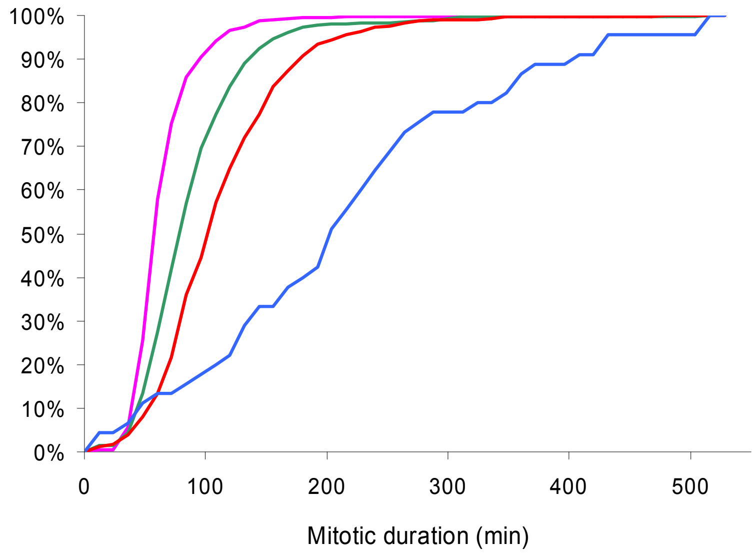

	HeLa H2B-GFP Treatment	N	median (min)	mean (min)	Comparison with DMSO treated cells Wilcoxon test *p* value
	DMSO	390	60	69.5	X
	Nocodazole 15nM	358	84	92.8	$\leq 2x10^{-16}$
	Nocodazole 25nM	299	108	116.8	$\leq 2x10^{-16}$
	Nocodazole 50nM	45	204	219.2	$2.22x10^{-16}$

Figure S2.
