# Supplementary material for: A Time-Series Method for Automated Measurement of Changes in Mitotic and Interphase Duration from Time-Lapse Movies
Source: PLoS One. 2011 Sep 26;6(9):e25511. doi: 10.1371/journal.pone.0025511 (PMC3180452; doi:10.1371/journal.pone.0025511)
Supplement: Figure S3 — The TSA approach detects dose-dependent increases in interphase duration upon treatment with higher doses of cycloheximide. HeLa H2B-GFP cells were treated with indicated concentrations of cycloheximide or untreated. Cumulative frequency curves of interphase duration for the cell populations are provided. The number of events in each sample (N), event duration median and mean as well as p-values for the Mann Whitney Wilcoxon statistical comparisons are provided. (PDF) [file pone.0025511.s003.pdf]

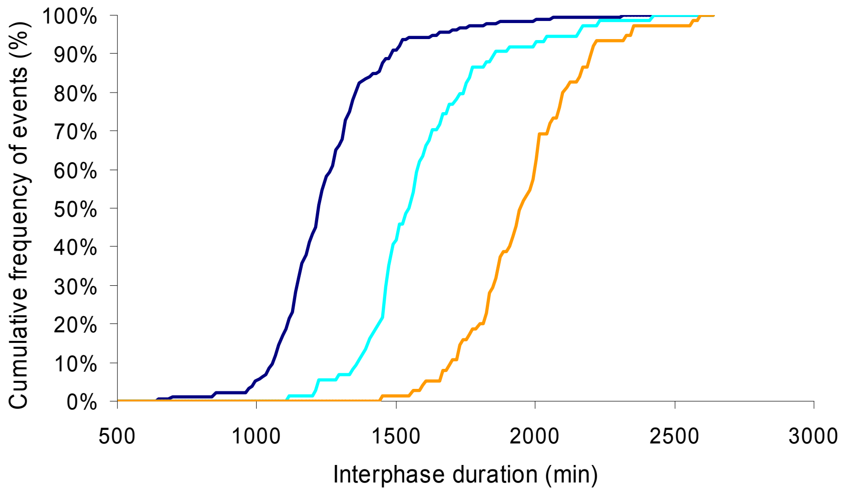

	HeLa H2B-GFP Treatment	N	Median (min)	Median (hrs)	Mean (min)	Mean (hrs)	Comparison with Untreated Wilcoxon test p-value
	Untreated	187	1224	20.4	1257.8	21.0	X
	Cycloheximide 0.05µg/mL	74	1554	25.9	1590.5	26.5	$\leq 2 \times 10^{-16}$
	Cycloheximide 0.1µg/mL	75	1956	32.6	1961.8	32.7	$\leq 2 \times 10^{-16}$

Figure S3.
